# Long-Term Responses of the Endemic Reef-Builder *Cladocora caespitosa* to Mediterranean Warming

**DOI:** 10.1371/journal.pone.0070820

**Published:** 2013-08-12

**Authors:** Diego K. Kersting, Nathaniel Bensoussan, Cristina Linares

**Affiliations:** 1 Departament d'Ecologia, Universitat de Barcelona, Barcelona, Spain; 2 IPSO FACTO, SCOPArl, Pôle Océanologie, Marseille, France; Universidade Federal do Rio de Janeiro, Brazil

## Abstract

Recurrent climate-induced mass-mortalities have been recorded in the Mediterranean Sea over the past 15 years. *Cladocora caespitosa,* the sole zooxanthellate scleractinian reef-builder in the Mediterranean, is among the organisms affected by these episodes. Extensive bioconstructions of this endemic coral are very rare at the present time and are threatened by several stressors. In this study, we assessed the long-term response of this temperate coral to warming sea-water in the Columbretes Islands (NW Mediterranean) and described, for the first time, the relationship between recurrent mortality events and local sea surface temperature (SST) regimes in the Mediterranean Sea. A water temperature series spanning more than 20 years showed a summer warming trend of 0.06°C per year and an increased frequency of positive thermal anomalies. Mortality resulted from tissue necrosis without massive zooxanthellae loss and during the 11-year study, necrosis was recorded during nine summers separated into two mortality periods (2003–2006 and 2008–2012). The highest necrosis rates were registered during the first mortality period, after the exceptionally hot summer of 2003. Although necrosis and temperature were significantly associated, the variability in necrosis rates during summers with similar thermal anomalies pointed to other acting factors. In this sense, our results showed that these differences were more closely related to the interannual temperature context and delayed thermal stress after extreme summers, rather than to acclimatisation and adaption processes.

## Introduction

Since the late 20^th^ century, global warming has been enhanced by human activities [1]. In this ongoing climatic change, climatic models predict that the Mediterranean Sea will be among the regions that are most affected by the warming trend and the increase of extreme events [2], [3]. In fact, warming trends in the last decades are well documented for the Mediterranean Sea, in both deep and coastal waters [4]–[7].

In the Mediterranean Sea, the frequency of abnormally warm summers has increased, resulting in unprecedented mass-mortality events. Although some early mortalities were detected in the 1970s and 1980s (e.g., [8], [9]), the first multispecies mass-mortality event was described in the NW Mediterranean in the summer of 1999 [10]–[12]. In the summer of 2003, a new mass-mortality episode occurred in NW Mediterranean coastal waters, this time over a larger geographic area [13]. Both events affected over 30 species of benthic invertebrates, mostly cnidarians, sponges and bryozoans [13], [14].

While the relationship of these mortalities to water temperature was unequivocal [11], [13], different factors, such as energetic constraints due to prolonged summer stratification of the water column [5] and pathogens [15], have been linked to the direct cause of death of the organisms. To date, several studies have examined the direct relationship between sea-water temperature and the mortality patterns of affected species; however, these studies are basically field studies encompassing one or a few years of observations [13], [16], [17] or laboratory experiments [5], [18]. Hence, there is an important lack of long-term studies assessing the long-term responses of temperate species to ongoing warming.

*Cladocora caespitosa,* the sole zooxanthellate scleractinian reef-builder in the Mediterranean, is among the organisms affected by these mortalities [12], [13], [19], [20]. Although it can be considered a conspicuous species, extensive bioconstructions of this endemic coral (i.e., banks; [21]) are very rare at the present time and are threatened by global change-related disturbances, such as the above-mentioned mortalities as well as the presence of invasive species [22], [23]. Although an important effort has been made to study the thermal tolerance of this species in aquaria [20], [24], [25], no study has assessed the long-term effects of warming-induced mortalities on natural *C. caespitosa* populations, especially on the endangered micro-reefs of this coral.

Here, we provide, for the first time, an analysis of the relationship between seawater warming and mortality in a *C. caespitosa* population over an 11-year period. We do so using data on the local water temperature regime for the period from 1991 to 2012 in the Columbretes Islands; this data set can also provide additional information on Mediterranean warming trends. The objectives of the present work are to study the existence of correlative evidence between the occurrence and intensity of the necrosis events and the local sea surface temperature (SST) regime and to compare the response of *C. caespitosa* throughout the recurrent mortality events in the Columbretes Islands to obtain information on the long-term effects of thermal anomalies on Mediterranean benthic species.

## Materials and Methods

### Ethics Statement

This study was conducted according to the permitting requirements of the Columbretes Islands Marine Reserve Authority (Secretaría General de Pesca, MAGRAMA). The Secretaría General de Pesca specifically issued the required permission for the *C. caespitosa* study in the Columbretes Islands Marine Reserve.

### Study site

**Figure 1:**
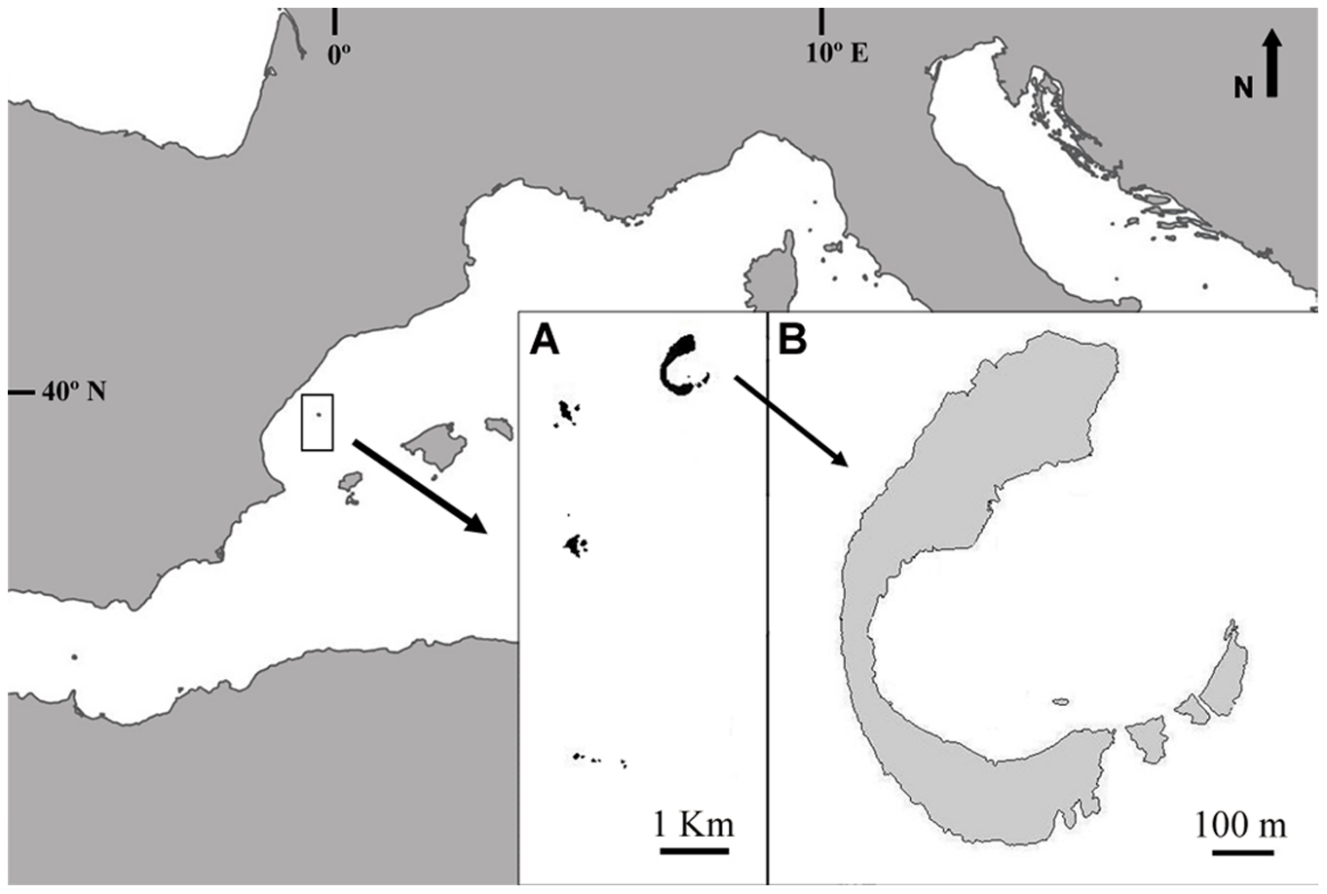
Map of the study site. A. The Columbretes Islands (NW Mediterranean, Spain). B. Illa Grossa Bay.

The Columbretes Islands emerge 30 nautical miles off the coast of Castelló (Spain, NW Mediterranean). A marine reserve encircles the archipelago covering an area of 5,500 ha. Illa Grossa (39°53.825′N, 0°41.214′E), the largest of the islets in the Columbretes (14 ha), is a C-shaped, drowned, Quaternary volcanic caldera (Fig. 1). The studied *C. caespitosa* population occurs in the central area of the bay formed by this islet (150,000 m^2^, 5–30 m depth range); with the highest coral cover values in the NW and SE parts. The cumulative coral cover in the bay reaches 2,900 m^2^ in a mixed bank-bed colony distribution [22].

### *C. caespitosa* mortalities

The impact of tissue necrosis on the *C. caespitosa* colonies was studied each year over the period 2002–2012. Mortalities were described and quantified by combining annual random transects and long-term monitoring of individually identified colonies. In the random transects, a total of 110 to 160 colonies were surveyed annually during the autumn (October – November). The long-term annual monitoring of identified colonies began in 2002 with 26 individually marked and mapped colonies; which were increased to 250 in 2006. The surveyed colonies occurred at a depth range of 5 to 20 m, and their maximum diameters ranged from 5 to 150 cm. Schemes and photographs of each colony were used in each survey to depict the areas affected by necrosis.

In each surveyed colony the following data were obtained: depth, percentage of the colony area affected by necrosis (in increments of 10% and differentiating recent or old necrosis) and the size of the colony through its maximum axis. The percentage of necrosis was always related to the living area of the colony. Necrosed areas below 10% were not considered to prevent confusion with other sources of natural mortality, such as those eventually induced by depredation by the gastropod *Babelomurex cariniferus* (Kersting DK, pers. obs.).

To detect delayed necrosis in the *C. caespitosa* colonies, additional surveys were undertaken four to five months after the first necrosis was detected.

Kolmogorov-Smirnov two-sample tests were used to determine whether there were significant differences in necrosis for the following comparisons: (i) along the depth gradient (5–15 m vs. 15–20 m; because vertical temperature profiles showed weak vertical gradients (<1°C) in the upper 15 m of depth during the warmest period), (ii) between the two main mortality periods (2003–2006 vs. 2008–2012) and (iii) during the second mortality period, between colonies that were previously unaffected (<10% necrosis) or affected (≥10% necrosis) during the first mortality period. This last test explored the existence of any degree of acclimatisation over time.

Kruskal-Wallis analysis was used to test for differences in necrosis depending on colony size (maximum diameter size classes: <25 cm, 25–50 cm, >50 cm).

### Temperature measurements

The SST data have been recorded daily in the Columbretes Islands Marine Reserve since 1991 at depths of 1 m using a calibrated mercury-in-glass thermometer (Thies Clima, model 2.2141.00.64, Göttingen, Germany). The temperature was measured between 8:00 and 9:00 a.m. by the Marine Reserve wardens following the same protocol (bucket sampling in the first meter of water and direct measurement with the thermometer). Overall, 6,028 daily measurements of SST were collected, which covers 75% of the 1991–2012 period, with a mean value of 274 data logs per year and a mean temporal cover of 87% during the summer (June-September). However, with only 27 data logs during the summer, the year 2000 was not considered in the statistical analyses.

Uncertainty in SST from bucket measurement is on the order of a few tenths of a degree C [26]. Comparisons with hourly records recorded by autonomous data loggers (Water Temp pro v2, ONSET, Cape Cod, MA, USA; accuracy: 0.21°C, resolution: 0.02°C) at 1 m depth from June 2011 to October 2012 yielded very good results, indicating that these punctual measurements reflected the near surface thermal environment (T_1m_  = 0.97 SST + 0.64, r = 0.99, p<0.001, N = 446). Additional temperature profiles (0–20 m) were recorded monthly in the Illa Grossa Bay from 2004 to 2007 using an SBE 39 temperature and pressure sensor (Sea-Bird Electronics, Bellevue, WA, USA). Since 2007, the bay was equipped with Stowaway Tidbits (ONSET, Cape Cod, MA, USA; accuracy: 0.2°C, resolution: 0.14°C) autonomous sensors set at depths of 5, 10, 15 and 20 m (1 hour data-sampling frequency). These sensors were installed in the same area as the permanent *C. caespitosa* transects.

Data obtained from the temperature profiles and the autonomous sensors were used to obtain information on the vertical gradients during the summer (June-September). Data from the autonomous sensor located at a depth of 15 m (average depth of the *C. caespitosa* population; [22]) were compared to the SST data for the summers from 2007 to 2012 to validate the use of the latter longer temperature series for the posterior necrosis-temperature correlation analyses (T_15m_  = 1.13 SST – 4.74 , r = 0.76, p<0.001, N = 678). Summer SST anomalies (i.e., the temperature obtained in the studied summer minus the average of the summers from the original data set (1991–2012)) were obtained for the studied summers (June-September, 2002–2012). Differences in summer SST anomalies among years were analysed using a one-way ANOVA and a Scheffé test for multiple comparison.

The persistence of high water temperatures during the studied summers was recorded as the number of days in which the SST exceeded certain temperature thresholds (from 24 to 28°C).

### Correlation between mortality and water temperature

Three mortality descriptors were selected to study the relationship between mortality events and SST anomalies: 1) The mean percentage of the coral's injured surface (hereafter, “necrosis”); 2) the percentage of colonies that were affected in their entirety by the necrosis (hereafter, “total mortality”) and 3) the percentage of colonies that were affected by the necrosis to some extent (hereafter, “affected colonies”).

Pearson's product-moment correlation was used to examine the relationship among the three descriptors (necrosis-affected colonies: r = 0.97, p<0.001; necrosis-total mortality: r = 0.81, p<0.005; affected colonies-total mortality: r = 0.70, p<0.05; N = 11). Necrosis was chosen as the principal mortality descriptor because its use has been generalised in previous mortality studies (e.g. [12], [13], [20], [27]–[29]).

The SST descriptors used were as follows: 1) summer SST anomalies and 2) persistence of temperature thresholds (i.e., the number of days over temperature thresholds 24, 25, 26, 27 and 28°C).

Multiple linear correlation analyses were performed to explore the relationship between the temperature and mortality descriptors. These analyses were performed for the whole studied period (2002–2012) and for the different mortality periods separately, in order to search for differences between them. The correlation analyses were also performed without the non-mortality years in order to study the role of the necrosis intensity in the correlation with the temperature descriptor.

## Results

### *C. caespitosa* mortalities: pattern of necrosis and inter-annual incidence

Old basal necrosis (i.e., accumulated necrosis prior to 2002) of approximately 3% was registered during the first colony surveys in 2002 and 2003. The first mass-mortality event affecting *C. caespitosa* was detected in September 2003. Recurrent mortalities were then detected at the end of the summers of 2004, 2005 and 2006. No mortality was detected in 2007. Although less virulent, necrosis events occurred again during every summer from 2008 to 2012.

**Figure 2:**
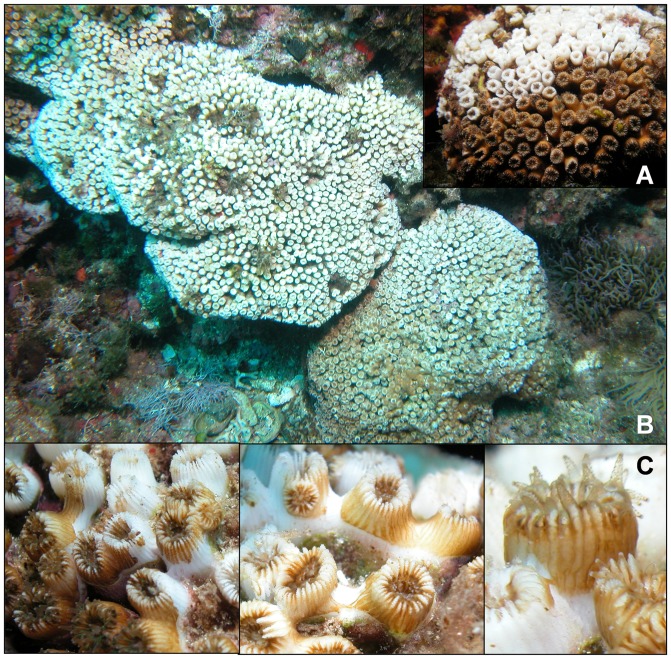
Mortality of *C. caespitosa*. A. *C. caespitosa* colony showing partial necrosis. B. Totally affected colony. C. The necrosis process in the polyps of *C. caespitosa*.

The polyp mortality was always characterised by direct tissue necrosis without massive loss of zooxanthellae (i.e., the polyps never lost the brownish-green colour given by the zooxanthellae). Tissue necrosis began at the basal part of the polyps; in these first stages, the polyps often remained expanded. Necrosis gradually affected polyp structure until all tissue disappeared, leaving the bare skeleton (Fig. 2). When colonies were only partially affected by necrosis, the dead polyps were always adjacent to each other, and the colony necrosis had a patched appearance. The evaluation of the accumulated occurrence of the necrosis patches in each colony showed that necrosis occurred both in the upper part and lower sides of the colony in 89.5% of all cases. The first signs of mortality were always detected during August and the beginning of September.

No delayed necrosis was ever detected, when the event was over, the necrosed areas of the colonies remained unchanged, and epibionts rapidly covered the damaged parts.

**Figure 3:**
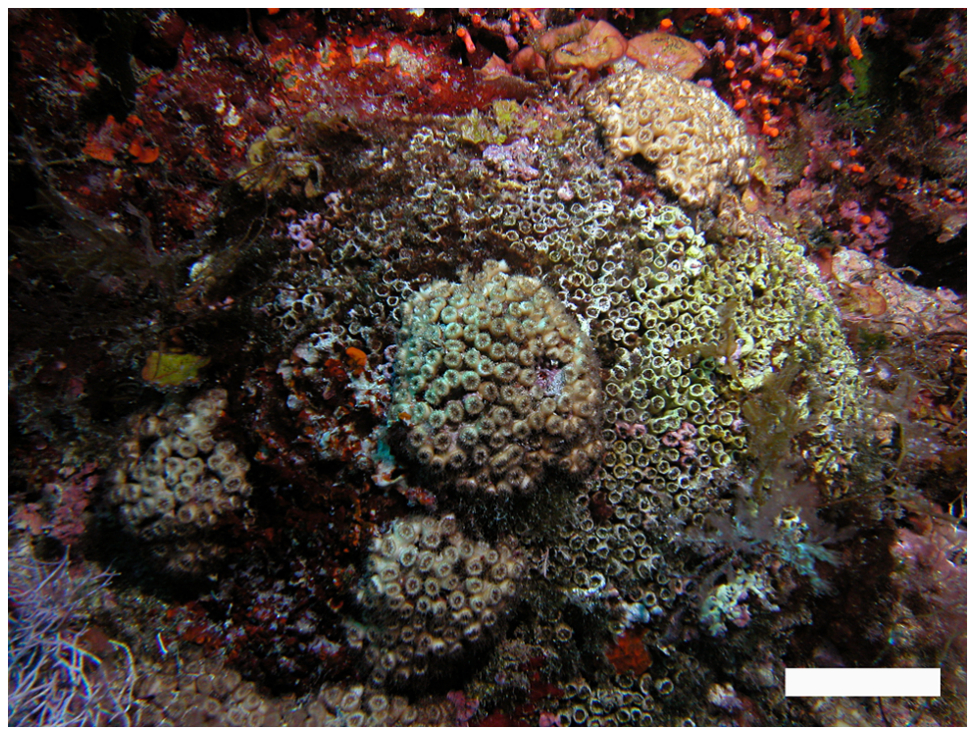
“Colony-on-colony” recruitment in a necrosis-affected *C. caespitosa* colony. Scale bar: 5 cm.

Recovery of these necrosed areas was never detected. However, in the last years of the survey (2010, 2011 and 2012), the recolonisation of dead colony areas was registered; this occurred through the recruitment of new *C. caespitosa* colonies on the old, dead polyps (Fig. 3). This colony-on-colony recruitment was recorded in 16.26% of the colonies that had experienced partial or complete mortality (average necrosis 80.60±20.3% (± SD)). Through this process, the recolonised colonies gained between 10 and 30% of new, living colony area.

Over the studied period, 80% of the monitored colonies (N = 250) were affected to some extent (partially or totally) by multiple mortality events. Considering the information from the fixed and random colony transects, the total colony area that was affected by the accumulated, recurrent necrosis was estimated to range between 55 and 80%.

**Figure 4:**
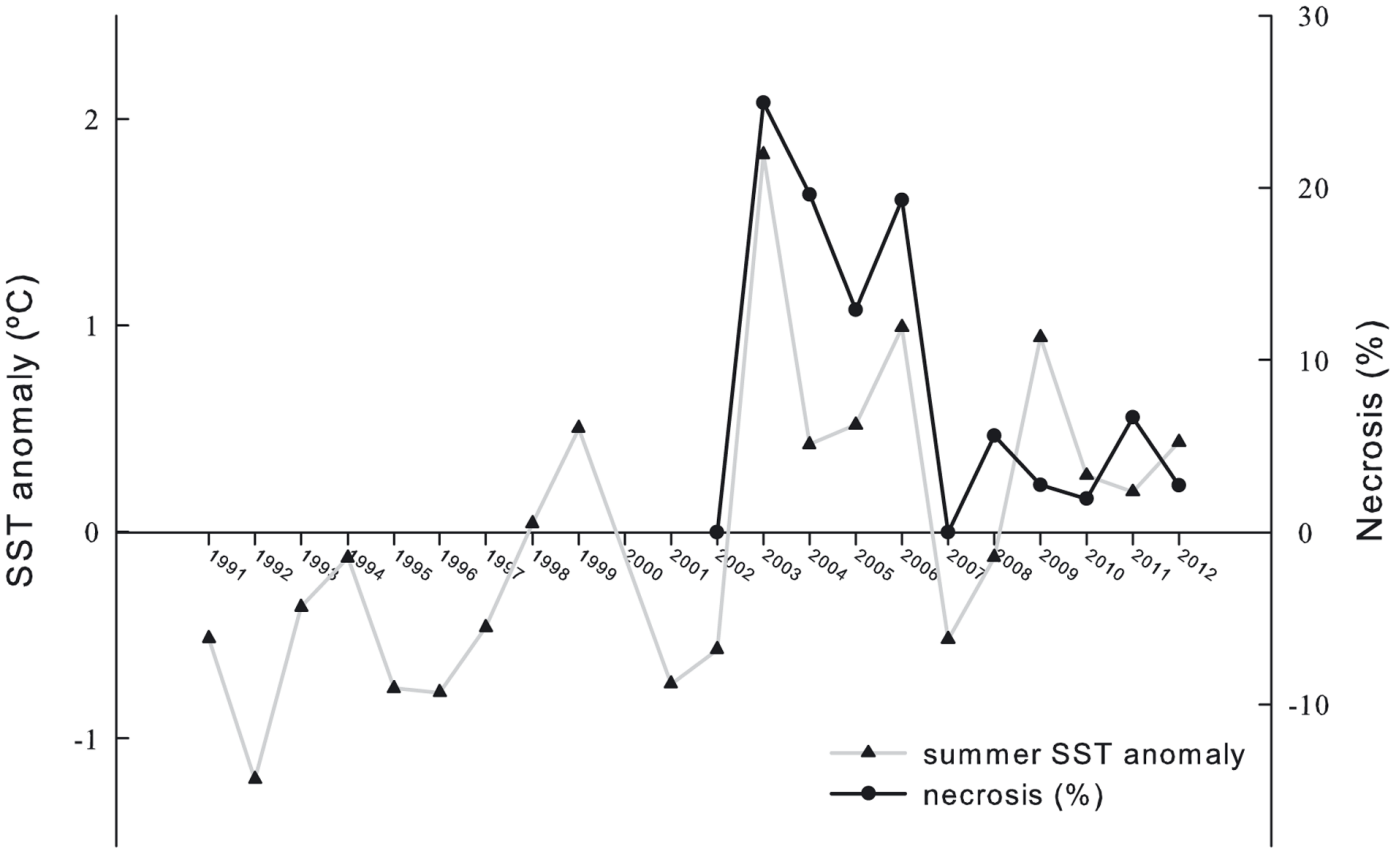
*C. caespitosa* necrosis rates (2002–2012) and summer SST anomalies (1991–2012).

The highest necrosis values were recorded during the 2003 event, during which 13.39% of the surveyed colonies died completely and necrosis reached an average of 25% (24.94±37.82%). Important mortalities occurred after the following summers (2004–2006), with necrosis values ranging between 12.91±27.46% and 19.62±29.49%. The recurrent mortality events that followed from 2008 to 2012 registered much smaller percentages of necrosis (ranging between 1.95±10.78% and 6.67±18.11%). See Figure 4 and Table 1. Generally, necrosis rates showed high variability between colonies, and affected and unaffected colonies were commonly found one beside each other.

**Table 1:** Mortality and temperature descriptors.

	Necrosis (% ± SD)	Affected colonies (%)	Total mortality (%)	SST anomaly (°C)	24°C	25°C	26°C	27°C	28°C
**2002**	0	0	0	−0.57	66	22	4	0	0
**2003**	24.94±37.82	46.43	13.39	1.83	98	82	61	44	25
**2004**	19.62±29.49	53.64	3.31	0.42	79	51	36	6	0
**2005**	12.91±27.46	26.36	5.43	0.52	85	64	33	0	0
**2006**	19.30±31.02	38.46	2.43	0.99	89	72	43	19	13
**2007**	0	0	0	−0.52	63	27	8	0	0
**2008**	5.61±18.50	12.34	0.43	−0.12	75	47	25	7	0
**2009**	2.76±10.26	11.69	0.43	0.94	90	75	61	22	11
**2010**	1.95±10.78	4.78	0.43	0.27	87	66	43	2	0
**2011**	6.67±18.11	17.47	0.87	0.19	86	52	27	8	3
**2012**	2.73±11.93	10.55	0	0.43	81	66	37	12	2

Note that necrosis is given in reference to the remaining living colony area.

Total mortality (100% of necrosed surface) was mostly due to a single mortality event rather than to accumulated necrosis from multiple, recurrent events. In this sense, 26.7% of the studied colonies experienced total mortality following a single event (half of these colonies died in 2003), while 6.9% experienced total mortality as a result of repeated necrosis events.

Significant differences were found in necrosis over the entire study period among the selected depth ranges (Kolmogorov-Smirnov test, p<0.001). In contrast, no significant differences were found between necrosis and colony size (Kruskal-Wallis test, p = 0.415).

**Figure 5:**
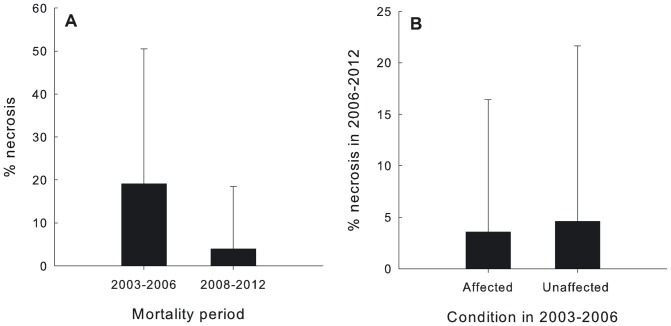
A. Percentage of necrosis (mean ± SD) detected in each mortality period. B. Percentage of necrosis (mean ± SD) detected in the second mortality period in colonies that were either affected or unaffected in the first mortality period.

The average percentage of necrosis was significantly higher during the first period than the second one: 19.07±31.45% between 2003 and 2006 vs. 3.96±14.52% between 2008 and 2012 (Fig. 5a; Kolmogorov-Smirnov test, p<0.01). In contrast, similar necrosis rates were recorded during the second period from colonies that were unaffected or affected during the first period (4.59±17.06% vs. 3.57±12.84%, respectively) (Fig. 5b; Kolmogorov-Smirnov test, p>0.1).

### Water temperature regime: annual cycle, warming trend and thermal anomalies

**Figure 6:**
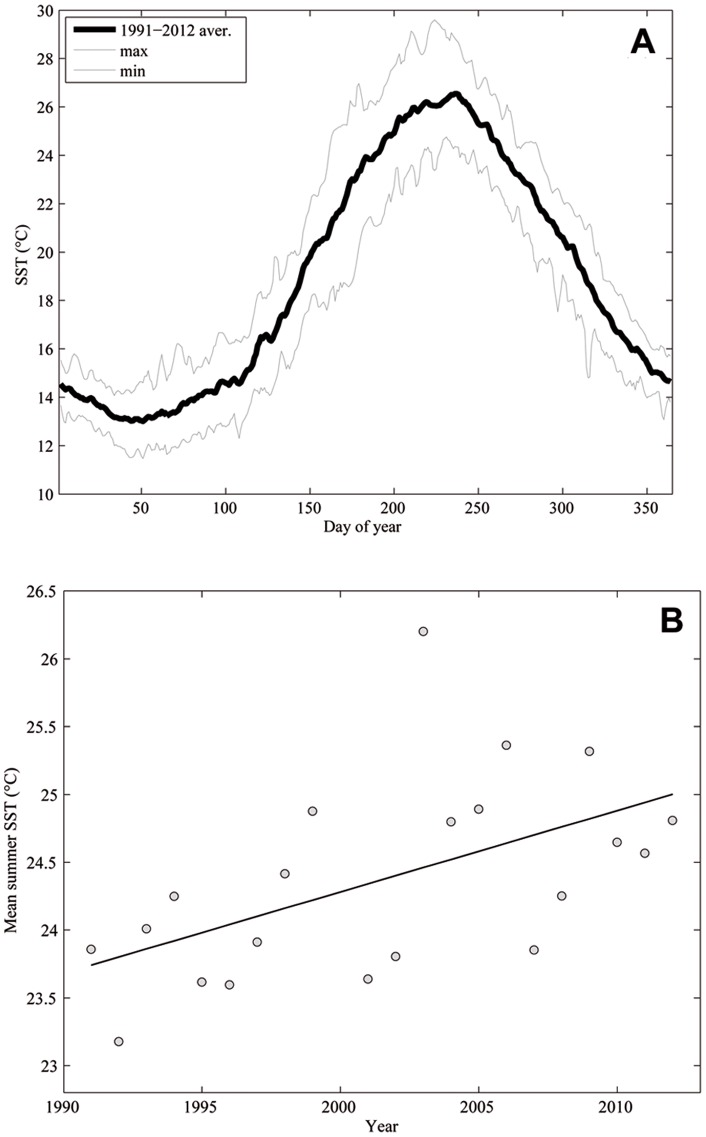
A. SST mean annual cycle in the Columbretes Islands (1991–2012). B. Mean summer SST (June-September, 1991–2012).

Annual cycles showed a minimum of ca. 12°C in mid-February and a maximum between 24.9 and 29.6°C in August (Fig. 6a). The seasonal warming typically had two phases: slow warming rates until mid-April, followed by steeper gradients through the end of June (0.19 vs. 0.87°C per week). SST cooling was observed from the end of August to the end of year at a rate of 0.66°C per week.

Over the period studied, SST exhibited a warming trend of 0.04°C per year (r = 0.30, N = 227). Focusing only on summer SST (June to September), the warming trend was even stronger, reaching 0.06°C per year (r = 0.55, N = 21) (Fig. 6b).

The frequency of positive thermal anomalies during the summer has increased markedly since 2003 (Fig. 4). In 1991–2002, all averaged summer thermal anomalies were negative, except in 1998 and 1999. Contrarily, in the second decade, positive anomalies were recorded during eight summers, occurring in two periods of four consecutive years and only interrupted by the 2007 and 2008 negative anomalies.

The summer SST anomalies varied significantly over time (one-way ANOVA, F_10, 1215_  = 14.802, p<0.001). The maximum significant differences were found when comparing 2003 with all but the warmest summers (i.e., 2006 and 2009). The summers with marked negative thermal anomalies (2002 and 2007) were significantly different from the warmest ones (Table S1).

**Figure 7:**
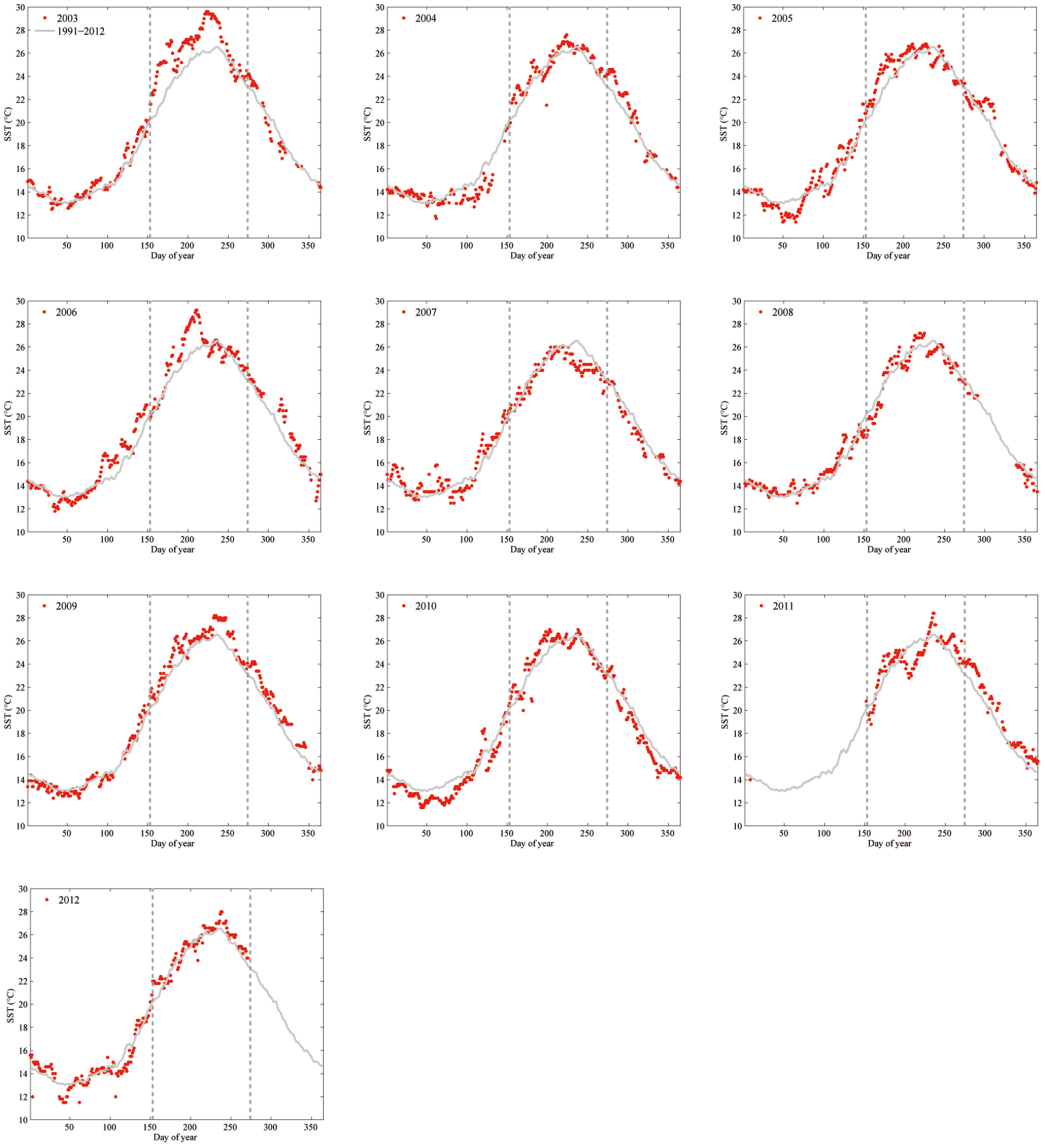
Annual thermal regime (2003–2012) and average SST for the data series 1991–2012. Dotted vertical lines delimit the summer period.

The summer of 2003 was the warmest of the 20-year-long SST data series, with an average positive anomaly of 1.83°C. During this summer, SST maxima of over 29°C were registered in the Illa Grossa Bay, and the average SST for the entire summer (June-September) was 26.20±2.06°C (Fig. 7). The following summers, i.e., 2004 and 2005, were characterised by moderate positive anomalies (0.42°C and 0.52°C, respectively). In the summer of 2006, high temperatures were reached again; temperature maxima were similar to those recorded in 2003, and an average anomaly of 0.99°C was registered. A second cycle of positive thermal anomalies began in 2009 and lasted until 2012. During these years, the maximum positive anomaly was reached in 2009 (0.94°C); positive anomalies were moderate in the summers of 2010, 2011 and 2012 (0.27°C, 0.19°C and 0.43°C, respectively). See Figs 4 and 7.

**Figure 8:**
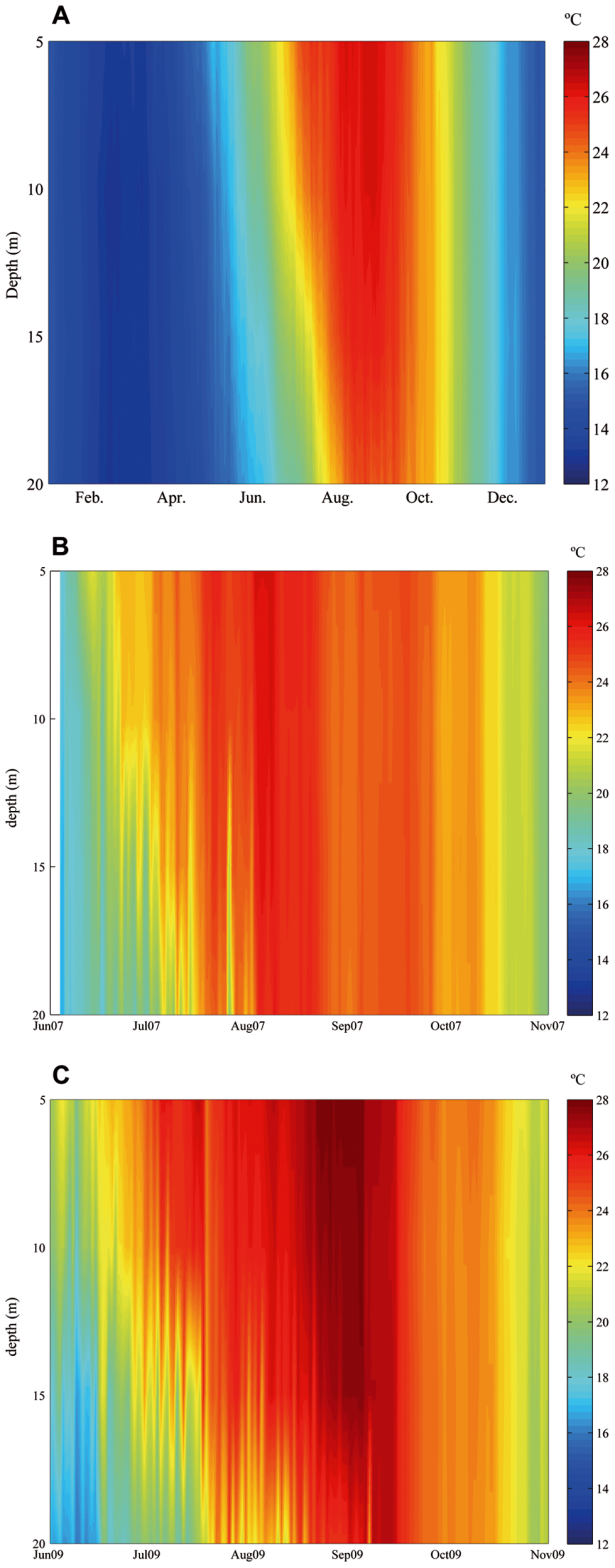
Thermal environment at a depth of 5 to 20 m. A. The 2007–2012 annual average B. Data from June to November in a summer with negative SST anomaly, 2007. C. Data from June to November in a summer with highly positive SST anomaly, 2009.

Average vertical temperature profiles attested to weak vertical gradients (<1°C) in the upper 15 m of the water column during the warmest period (August) and in the upper 10 m over the entire summer (Fig. 8).

In the years with available data (2004–2012), water temperatures remained over 25°C at depths of 15 m at least during August. The only year without mortality during this time span (2007) had 10 weeks over 24°C, 3 weeks over 25°C and 0.3 weeks over 26°C at 15 m (Fig. 8b). In the years with mortality, water temperatures at depths of 15 m remained over 25°C for between 5 and 10 weeks (Fig. 8c).

### Correlation between mortality and water temperature

Necrosis and SST anomalies showed a significant positive correlation over the entire studied period (2002–2012; r = 0.75, p<0.01) (Table S2). Similarly, the other mortality descriptors also showed a positive relationship with SST anomalies (total mortality, r = 0.75, p<0.01; affected colonies, r = 0.70, p<0.05).

When performing the analyses with the two mortality periods separately, the relationship between mortality descriptors and SST anomalies was highly correlated during the first period (necrosis-SST anomalies, r = 0.94, p<0.01) but lost significance during the second period. If the non-mortality years (2002 and 2007) were not considered, the correlation between these variables over the entire studied period lost significance (Table S2).

The correlation between necrosis and persistence of temperature thresholds over the whole studied period was significant only for the warmest limits (necrosis-27°C, r = 0.61, p<0.05; necrosis-28°C, r = 0.63, p<0.05), while during the first mortality period the correlation was significant for the colder thresholds (necrosis-24°C, r = 0.93, p <0.01; necrosis-25°C, r = 0.92, p<0.01; necrosis-26°C, r = 0.97, p<0.01). No correlation between necrosis and persistence of temperature thresholds was found when analyzing the second period separately (Table S2).

## Discussion

Historically, mass coral bleaching has been linked to episodes of thermal stress in tropical corals; this is an increasing concern around the world (see [30] for a review). Nonetheless, monitoring the mortalities in the temperate scleractinian reef-builder *C. caespitosa* in the Columbretes Islands (NW Mediterranean Sea) over an 11-year period allowed describing, for the first time, the relationship between recurrent mortality events and local SST regimes in the Mediterranean Sea.

### Patterns of mortality

The observed necrosis process in the Columbretes Islands was very similar to previous descriptions of *C. caespitosa* necrosis in the Ligurian Sea [20]. In accordance with previous studies based on field and laboratory data, *C. caespitosa* polyps died due to progressive tissue necrosis with no signs of zooxanthellae loss [20], [25], [31]. The absence of bleaching is most likely related to the resistance to increases in temperature shown by the *Symbiodinium* (clade temperate-A, [32]) in symbiosis with *C. caespitosa* [24].

Tissue regeneration after mortality episodes was not detected in the Ligurian Sea [20] or in the present study. This could be due to the phaceloid morphology of *C. caespitosa* colonies, built up by independent polyps, which makes the regeneration of adjacent damaged tissue by unaffected polyps difficult [20]. Conversely, the autonomy of the *C. caespitosa* polyps could also be responsible for the lack of delayed necrosis following mortality events as well as the lack of correlation between colony size and necrosis, as has been detected in temperate gorgonians [27]–[29]. Unexpectedly, although tissue recovery was not observed, another indirect but non-trivial mechanism of colony recovery was detected during the last years of the study. *C. caespitosa* recruits settled on the newly available space on the dead colony parts.

Decreases in necrosis rates with depth have been described for species living at greater depths than *C. caespitosa*, e.g., the gorgonian *P. clavata* [29]. Although the depth range of the studied *C. caespitosa* colonies places them above the thermocline depth during most of the summer, the relationship between necrosis and depth was consistent with the fact that the summer conditions begin sooner for shallower colonies because the thermocline typically reaches a depth of 15 m at the beginning of August. Therefore, *C. caespitosa* colonies living at shallower depths were more exposed to thermal stress and showed greater mortality rates. As a result, changes in the depth distribution of this population are expected in the future due to the disappearance of the shallower colonies.

### Relationship between mortality and temperature

Mortalities were recorded in the context of regional warming and occurred concomitantly with a shift in the regime of positive thermal anomalies in the Columbretes Islands. In particular, the first mortality was triggered by exceptionally warm conditions accompanied by the persistence for several days of extreme (>28°C) temperatures.

However, it is worth mentioning that our results are not in concordance with those found in the laboratory. During different aquaria thermo-tolerance experiments with *C. caespitosa* polyps (collected in the Ligurian Sea), the first signs of necrosis were detected after 5–7 weeks at 24°C, and all polyps that were exposed at 26°C and 28°C died after the treatments [20], [25]. Based on these experiments, the authors proposed that *C. caespitosa* is living close to its thermal limit during the summer period in the Ligurian Sea and a long-term increase at 24°C or above could be lethal for it. In the Columbretes Islands, water temperatures at 15 m remained over 24°C for 10 weeks during the summer of 2007, which recorded negative thermal anomaly. This time span was 3 to 5 weeks longer than that reported in the mentioned experiments and no necrosis was detected. Similarly, in the summer of 2009, the average extent of necrosis was approximately 3%, and *C. caespitosa* colonies at 15 m were exposed to temperatures greater than 24°C for 68 days and to temperatures greater than 26°C for 34 days; this exposure was approximately three times longer than the exposure that caused necrosis in 100% of the polyps in the aquaria experiment [25]. The differences found between the mortalities in aquaria (Ligurian Sea) and *in situ* (Columbretes Islands) could be related to two major points: differences in the thermal acclimatisation of *C. caespitosa* between both sites, taking into account that the colonies are naturally subjected to different thermal regimes, and the fact that aquaria experiments can only partially simulate the natural environmental conditions.

Another striking result is that the response of *C. caespitosa* to summers with positive thermal anomalies changed between the two mortality periods and particularly in relation to temperature thresholds. The correlation between necrosis and the persistence of water temperature thresholds for the entire data series was only significantly positive when assessed using the 27°C and 28°C threshold. However, a significant positive correlation between necrosis and temperature thresholds of 24°C, 25°C and 26°C was found when considering only the first mortality period, while no correlation was found for the second period.

During this 11-year study, mortality events occurred in two separated periods, i.e., 2003–2006 and 2008–2012. The average necrosis diverged significantly in these two periods (19% vs. 4%, respectively), and important differences in the average thermal anomaly were also found (1.00°C and 0.39°C, respectively). However, with the same positive thermal anomaly (approximately 1°C), different years such as 2006 and 2009 registered contrasting necrosis (19% vs. 3%, respectively).

As our results prove, it is unequivocal that sea water temperature is one of the main factors that triggered *C. caespitosa* mortality events. Nevertheless, the differences found in necrosis between years with similar thermal anomalies show that other factors are also acting in this process.

### Synergies with other factors

Water quality and ecosystem conservation has been ensured in the Columbretes Islands Marine Reserve since its creation in 1990. Furthermore, the location of the islands far from mainland (60 Km) guarantees low interaction with nearshore waters. Therefore, factors such as water quality or dysfunctions in trophic interactions derived from overfishing, that might be relevant in unprotected areas [33], were excluded in the present study.

Although irradiance, especially photosynthetically active radiation (PAR), has been shown to be directly related to tropical coral bleaching [34]–[36], we disregarded it as a possible factor acting in the *C. caespitosa* mortalities. Depending on the depth and water type, irradiance can be significantly attenuated [34], [37]. Bearing in mind the depth range of our studied *C. caespitosa* population we can assume an important reduction in irradiance. Furthermore, the zooxanthellae in symbiosis with *C. caespitosa* (*Symbiodinium* Clade A) are considered light-adapted [38], [39]. Finally, a pattern in the necrosis scars related to the effects of irradiance, as reported in tropical corals [34], was not observed in *C. caespitosa*.

Disease outbreaks have affected an increasing range of marine organisms in different geographic regions worldwide [40]. In the Mediterranean Sea, thermally dependent pathogens have been considered co-responsible for mass-mortalities and coral bleaching [15], [41], [42]. Although, as far as we know, no studies have dealt with this issue in *C. caespitosa*, the type of necrosis (lysis) suffered by this species could be related to a disease, such as that caused by *V. coralliilityticus*, which synthesises a potent extracellular protease that lyses coral tissue [43]. Although no analyses were conducted to detect opportunistic pathogens in the *C. caespitosa* mortalities, the possible role of pathogens or even polymicrobial consortiums as recently suggested in other tropical coral species [44], should not be disregarded. Previous studies have demonstrated that the occurrence of *Vibrio* bacteria in the NW Mediterranean Sea is climate linked, greatly increasing under the inﬂuence of positive temperature anomalies as the observed ones in Columbretes Islands [45].

In tropical corals, greater energy reserves or greater access to resources could compensate for decreased photosynthesis during bleaching events [46], [47]. In the Mediterranean Sea, temperature-related mortalities have been associated with physiological stress due to energetic constraints [5]. According to these data, Crisci *et al.* [17] considered physiological status to be a primary factor explaining differential mortality rates.

*C. caespitosa* has the ability to upregulate heterotrophy and maintain symbiosis, even under suboptimal conditions [48]. These authors detected maximum feeding effort when colonies were kept under high light with an irregular food source (typical Mediterranean summer conditions). Consequently, variation in the availability of food previous to and during warm summers could have an important effect in the energy budget of *C. caespitosa*. Furthermore, the impact of extreme summers (like 2003) on the energy budget of the polyps could be responsible for delayed effects in their physiological status.

Processes such as spawning that cause a reduction in tissue lipid content could also have an important effect on the severity of mortality [46]. Histological analyses showed that maximum gonadal development in *C. caespitosa* is reached in August [49] in coincidence with SST maxima, and spawning occurs at the end of the summer. Consequently, the interaction between sexual reproduction and necrosis could be reciprocal: necrosis could be enhanced due to increased energy investment in gonad development, and spawning could be affected by the mortality of the polyps.

With this in mind, we hypothesise that delayed physiological thermal stress could be the primary factor, acting together with temperature, that would explain the differences in necrosis during summers with similar thermal anomalies but with different interannual contexts. This sensitisation hypothesis has also been mentioned in regards to the mass-mortality of 1999 [11].

### Searching for acclimatisation and adaption processes

The processes of acclimatisation (phenotypic response) and adaption (genotypic response) have been extensively studied and discussed in relation to thermal anomalies causing bleaching events in tropical corals [30], [50]–[54]. While some authors extend hope for rapid evolution and adjustment [50], [51], others question the capacity of corals to adapt to rapid climate change [53].

Through comparisons of bleaching events in tropical corals, several authors have found that corals were more resistant to temperature stress as the bleaching events repeated [55]–[57] and that the bleaching resistance shown by corals at sites dominated by high-frequency SST variability could be a consequence of rapid directional selection following an extreme event [57].

Although the SST series in the Columbretes Islands showed a dramatic increase in the frequency of positive thermal anomalies, as well as a positive warming trend, the differences in mortality detected between summers with similar thermal anomalies did not seem related directly to directional selection. *C. caespitos*a colonies that survived the first mortality period were affected in the second period, although the thermal anomalies had lower positive values on average; therefore, survival was most likely not solely a result of differential survival of more tolerant genotypes.

In this sense, we found that necrosis in the second mortality period (2008–2012) showed no differences between colonies that were unaffected or affected during the first mortality period. Differences between these groups would have been expected if selection was acting on thermal tolerance.

Nevertheless, it is remarkable that approximately 20% of the surveyed colonies remained unaffected over the entire study period and that a very low percentage experienced total mortality due to accumulated recurrent necrosis. These results may indicate the occurrence of more tolerant colonies or even parts of colonies; however, as discussed above, selection for thermal tolerant genotypes alone cannot explain the detected changes in necrosis. In conclusion, these mortalities do not relate to previous necrosis impacts on the same colonies; the occurrence of necrosis at the colony level seems more closely related, in general terms, to random processes involving the occurrence of pathogens or the energetic status of the polyps, as previously discussed.

### The importance of context-dependent effects

The summer of 2003 was likely the warmest summer in Europe since 1500 [58] and affected 25 rocky benthic macroinvertebrate species over several thousand kilometres of Mediterranean coastline [13]. The mean SST anomaly registered in the summer of 2003 in the Columbretes Islands (1.83°C) was 80% warmer than the second positive SST anomaly recorded in the series (in 2006). During this summer, 25% of the area covered by *C. caespitosa* in the Columbretes Islands was necrosed.

As discussed above, the extreme conditions of 2003 could have been responsible for a delayed physiological stress in the colonies, influencing the mortalities registered in the following summers (2004 and 2005), which were quite important (approximately 20% and 13% of necrosis, respectively); however, the positive SST anomalies during these summers were relative low (0.42 and 0.52°C, respectively).

The second mortality period (2008–2012) began after a year with negative SST anomalies and no necrosis (2007). This could have given *C. caespitosa* enough rest to withstand the mortality events of the next summers with much lower necrosis, in addition to the fact that no extreme conditions (such as those observed in 2003) were present during the second period. In this period, summers with similar or even higher SST anomalies than in the first period exhibited mortality events with less than 7% necrosis. Although the first mortality event of the second period (2008) was registered after a summer with an average negative SST anomaly (−0.12°C), several weeks of strong positive anomalies were recorded during the middle of this summer.

However, what could have happened prior to 2002? The mortality of 2003 could be considered the first mass-mortality of *C. caespitosa* in the Columbretes Islands in the last two decades. Although necrosed colonies or sections of colonies were eventually covered by epibionts, they were perfectly noticeable over many years. Thus, a mass-mortality event prior to 2003 should have left a high percentage of detectable bare skeletons in the colonies, but the old necrosis detected was near 3%. This is consistent with the thermal anomalies recorded in the available SST series during the first decade of record (1991–2002), which were much lower and less frequent than during the second decade. The summer of 1999 could have been the one in which some mortality would have been expected because the SST anomaly reached 0.50°C; furthermore, this summer triggered a multispecies mass-mortality event in the NW Mediterranean [10], [12], [28]. That the summer of 1999 most likely did not cause high necrosis rates reinforces our hypothesis that some type of sensitisation or delayed stress occurred after the summer of 2003 because the summers of 2004 and 2005 had similar SST anomalies to those recorded for 1999 but triggered high necrosis.

Three important findings can be highlighted from the results obtained in this study. First, a significant positive correlation between mortality descriptors and SST anomalies was found over the entire studied period. Second, significant differences between the two mortality periods were found when correlation analyses were performed separately. Third, when removing the years without mortality (2002 and 2007) significance disappeared for the whole studied period. Two main conclusions can be drawn from these results. First, the significant, strong association between mortality descriptors and SST anomalies, when looking at the whole series, is more closely related to the concurrence of necrosis events and SST anomalies than to the specific intensity of these variables; as significance is lost when removing the years with no mortality and necrosis was generally detected in years with a positive SST anomaly, but summers with similar SST anomalies showed different responses in *C. caespitosa* necrosis. Second, the effects of the intensity of the SST anomalies on the necrosis rates seem to have been enhanced during the first period, which would be consistent with the delayed thermal stress hypothesis.

The complexity of the factors influencing these mortalities highlights the need for precise and continuous long-term monitoring of biotic and abiotic factors to move forward in our understanding of these events and their effects on the future viability of the benthic communities threatened by the increase in frequency and persistence of extreme events projected for the 21^st^ century in the Mediterranean [2], [3]. Recurrent extraordinary mortality episodes, such as the ones registered between 2003 and 2006, could likely be repeated and will threaten this species, which, due to its slow dynamics, will most likely not be able to cope with elevated mortality rates. Nevertheless, considering the less virulent mortalities registered in the second mortality period, the high coral cover in areas such as the Columbretes Islands [22], and the potential for colony-on-colony recruitment as an indirect mechanism of recovery, there can still be some hope for *C. caespitosa* banks in the Mediterranean Sea and particularly in the Columbretes Islands.

## Supporting Information

Table S1 **Scheffé's contrast test obtained from a one-way ANOVA comparing summer SST anomalies among years.**
(DOC)

Table S2 **Results of the multiple correlation tests between annual necrosis and temperature descriptors.** Significant correlation is highlighted in bold.* Without 2002 and 2007.
(DOC)
