# Supplementary material for: Long-Term Responses of the Endemic Reef-Builder Cladocora caespitosa to Mediterranean Warming
Source: PLoS One. 2013 Aug 12;8(8):e70820. doi: 10.1371/journal.pone.0070820 (PMC3741371; doi:10.1371/journal.pone.0070820)
Supplement: Table S1 — Scheffé's contrast test obtained from a one-way ANOVA comparing summer SST anomalies among years. (DOC) [file pone.0070820.s001.doc]

**Table S1.** Scheffé’s contrast test obtained from a one-way ANOVA comparing summer SST anomalies among years.

|  | **2002** | **2003** | **2004** | **2005** | **2006** | **2007** | **2008** | **2009** | **2010** | **2011** |
| --- | --- | --- | --- | --- | --- | --- | --- | --- | --- | --- |
| **2002** |  |  |  |  |  |  |  |  |  |  |
| **2003** | **<0.001** |  |  |  |  |  |  |  |  |  |
| **2004** | 0.100 | **<0.05** |  |  |  |  |  |  |  |  |
| **2005** | **<0.05** | **<0.05** | 1.000 |  |  |  |  |  |  |  |
| **2006** | **<0.001** | 0.424 | 0.928 | 0.970 |  |  |  |  |  |  |
| **2007** | 1.000 | **<0.001** | 0.177 | 0.087 | **<0.001** |  |  |  |  |  |
| **2008** | 0.967 | **<0.001** | 0.923 | 0.826 | 0.063 | 0.988 |  |  |  |  |
| **2009** | **<0.001** | 0.254 | 0.959 | 0.986 | 1.000 | **<0.001** | 0.081 |  |  |  |
| **2010** | 0.304 | **<0.001** | 1.000 | 1.000 | 0.621 | 0.445 | 0.994 | 0.699 |  |  |
| **2011** | 0.497 | **<0.001** | 1.000 | 0.998 | 0.441 | 0.646 | 0.999 | 0.517 | 1.000 |  |
| **2012** | 0.077 | **<0.05** | 1.000 | 1.000 | 0.928 | 0.144 | 0.904 | 0.960 | 1.000 | 1.000 |
