# Supplementary material for: Long-Term Responses of the Endemic Reef-Builder Cladocora caespitosa to Mediterranean Warming
Source: PLoS One. 2013 Aug 12;8(8):e70820. doi: 10.1371/journal.pone.0070820 (PMC3741371; doi:10.1371/journal.pone.0070820)
Supplement: Table S2 — Results of the multiple correlation tests between annual necrosis and temperature descriptors. Significant correlation is highlighted in bold.* Without 2002 and 2007. (DOC) [file pone.0070820.s002.doc]

**Table S2.** Results of the multiple correlation tests between annual necrosis and temperature descriptors. Significant correlation is highlighted in bold.* Without 2002 and 2007.

|  | **2002-2012 (N = 11)** | **2002-2007 (N = 7)** | **2007-2012 (N = 6)** | **2002-2012* (N = 9)** |
| --- | --- | --- | --- | --- |
| **Necrosis - SST anomaly** | **r = 0.746, p<0.01** | **r = 0.939, p<0.01** | r = 0.161, p = 0.761 | r = 0.647, p = 0.060 |
| **Necrosis – 24 ºC** | r = 0.582, p = 0.061 | **r = 0.928, p<0.01** | r = 0.404, p = 0.428 | r = 0.399, p = 0.2875 |
| **Necrosis – 25 ºC** | r = 0.536, p = 0.090 | **r = 0.923, p<0.01** | r = 0.151, p = 0.775 | r = 0.271, p = 0.480 |
| **Necrosis – 26 ºC** | r = 0.517, p = 0.104 | **r = 0.974, p<0.01** | r = 0.059, p = 0.912 | r = 0.257, p = 0.505 |
| **Necrosis – 27 ºC** | **r = 0.607, p<0.05** | r = 0.750, p = 0.086 | r = 0.232, p = 0.658 | r = 0.513, p = 0.158 |
| **Necrosis – 28 ºC** | **r = 0.629, p<0.05** | r = 0.695, p = 0.125 | r = 0.063, p = 0.906 | r = 0.581, p = 0.101 |
